# Serum trimethylamine-*N*-oxide and protein energy wasting: some factors that should be considered

**DOI:** 10.1080/0886022X.2023.2165446

**Published:** 2023-02-08

**Authors:** Wenyun Wang, Chengji Zhao, Gang Li, Binde Li, Yanan Wang, Yongfeng Lao, Rongxin Li, Zhilong Dong

**Affiliations:** a Department of Pediatric Surgery, Second Hospital of Lanzhou University, Lanzhou, PR China; b Department of Second College of Clinical Medicine, Lanzhou University, Lanzhou, PR China; c Department of Urology, Second Hospital of Lanzhou University, Lanzhou, PR China

Dear Sir,

We have read with great interest the paper entitled ‘Correlation between serum trimethylamine-*N*-oxide concentration and protein energy wasting in patients on maintenance hemodialysis’ by Hu et al. [1]. The cross-sectional study comprised 50 healthy individuals and 93 chronic kidney disease (CKD) patients undergoing maintenance hemodialysis (MHD) to analysis the correlation between CKD-associated protein energy wasting (PEW) and serum trimethylamine-*N*-oxide (TMAO) levels. The serum TMAO were 5653.76 ± 2853.51 ng/mL in the MHD group and 254.92 ± 197.88 ng/mL in the control group (*p* <  .01) by using a liquid chromatography–tandem mass spectrometry method. The MHD patients were divided into two groups by the median level: the high TMAO group (7206 ng/mL) with 85% in PEW and the low TMAO group (4080.9 ng/mL) with only 34% in PEW. The linear regression analysis showed that higher serum TMAO was negatively correlated with BMI (*r* = –0.343, *p* <  .001) and dietary protein intake (*r* =  −0.469, *p* <  .01), which are indicators of PEW. Multivariate logistic regression showed that the prevalence of PEW increased with every 100 ng/mL increase in the TMAO level in model 3 (OR  =  1.09, 95% CI 1.05–1.13, *p* =  .001), which is displayed in Table 3. It is novel to analyze the correlation between CKD-associated PEW and serum TMAO levels. However, we pay special attention to the several key clinical indicators of this research because it caused us some confusion.

First, residual renal function (RRF): TMAO is a 75 Da metabolite that is an enterogenous micromolecular toxin with mainly derived metabolite of l-carnitine and choline by the gut microflora, and rapidly cleared by the kidneys through a combination of glomerular filtration and tubular secretion [2]. According to the former study, it showed that serum TMAO levels in HD patients reflect the net effect of TMAO production and clearance. HD clears TMAO at a rate similar to creatinine, with re-accumulation to serum concentrations of approximately 100 μM before the subsequent dialysis session [2,3]. However, the present study by Hu et al. [1] did not assess RRF which is a major contributor to TMAO clearance.

Hai et al. [3] found that the production rate of TMAO in MHD patients with 606 ± 220 μmol/day was not different than the urinary excretion of the normal subject with 533 ± 248 μmol/day (*p* >  .05). Pre-dialysis plasma levels of TMAO were markedly higher than in the normal subjects at 77 ± 26 μM (*p* <  .05). The TMAO for clearance of dialysis in MHD patients and clearance of urinary in normal control were 165 ± 72 mL/min and 219 ± 78 mL/min, respectively. An observational prospective cohort study to explore renal excretion and mechanisms of accumulation of TMAO during CKD showed that measured GFR (mGFR) was negatively correlated to plasma TMAO (*r*^2^ = 0.388, *p* <  .0001). A subgroup of 32 patients underwent urinary clearance tests for TMAO, which was not significantly different from mGFR, with a mean ± SD TMAO fractional excretion of 105%±32%. This means a complete renal excretion of TMAO by glomerular filtration [4].

Second, medications: an observational cohort study of 423 ESRD patients (included 261 with HD) by Dai et al. [5] showed that a positive correlation between sevelamer use with TMAO (*r*  =  0.19, *p* <  .05) in univariate Spearman’s rank correlation analysis. Multivariate linear regression analysis showed the significant and independent association between poor vitamin K status and high serum TMAO (coefficient 0.19, *p* =  .006, model *R*^2^ = 0.13) in HD patients. It found that dysbiosis in CKD due to other iatrogenic factors are the results of iron therapy, proton pump inhibitors, and antibiotics [6]. A systematic review by Kalagi et al. [7] showed that broad-spectrum antibiotics (including metronidazole and ciprofloxacin), nonsteroidal anti-inflammatory drug aspirin, fat-soluble vitamins (vitamin D3), and water-soluble vitamins (B vitamins) showed a significant effect on lowering plasma TMAO; however, metformin and of l-carnitine supplementation were found to significantly increase TMAO concentrations.

Besides this, biomarkers of inflammation. Missailidis et al. [8] performed an observational prospective cohort study in 80 controls and 179 CKD3–5 patients (CKD 3: *n*  =  30; CKD4: *n*  =  28; CKD5: *n*  =  116) showed that the correlations were observed between TMAO and IL-6 (*r*  =  0.42, *p* <  .0001) and hsCRP (*r*  =  0.17, *p* =  .022). Subgroup analysis showed the comparative analysis of inflamed (hsCRP ≥ 10 mg/L, *n*  =  38) and non-inflamed patients (hsCRP  <  10 mg/L, *n*  =  141), demonstrated higher TMAO levels (*p* <  .002) in inflamed patients. A case-control study included 88 HD patients with PEW (*n*  =  22, the seven-point subjective global assessment (SGA) scores 1–5) and normal nutritional status (*n*  =  66, the seven-point SGA scores 6–7) who were matched 1:3 for age and sex, it showed that patients with PEW had a significantly higher plasma levels of IL-6 (*p* =  .004) and TNF-α (*p* =  .003), but significantly lower body weight (*p* =  .002), lower body mass index (*p* =  .001), a decreased α-diversity (observed species, Chao 1, Shannon, and Simpson) and a reduced abundance of butyrate-producing bacteria than patients with normal nutritional status (*p* <  .05) [6].

**Figure 1.**
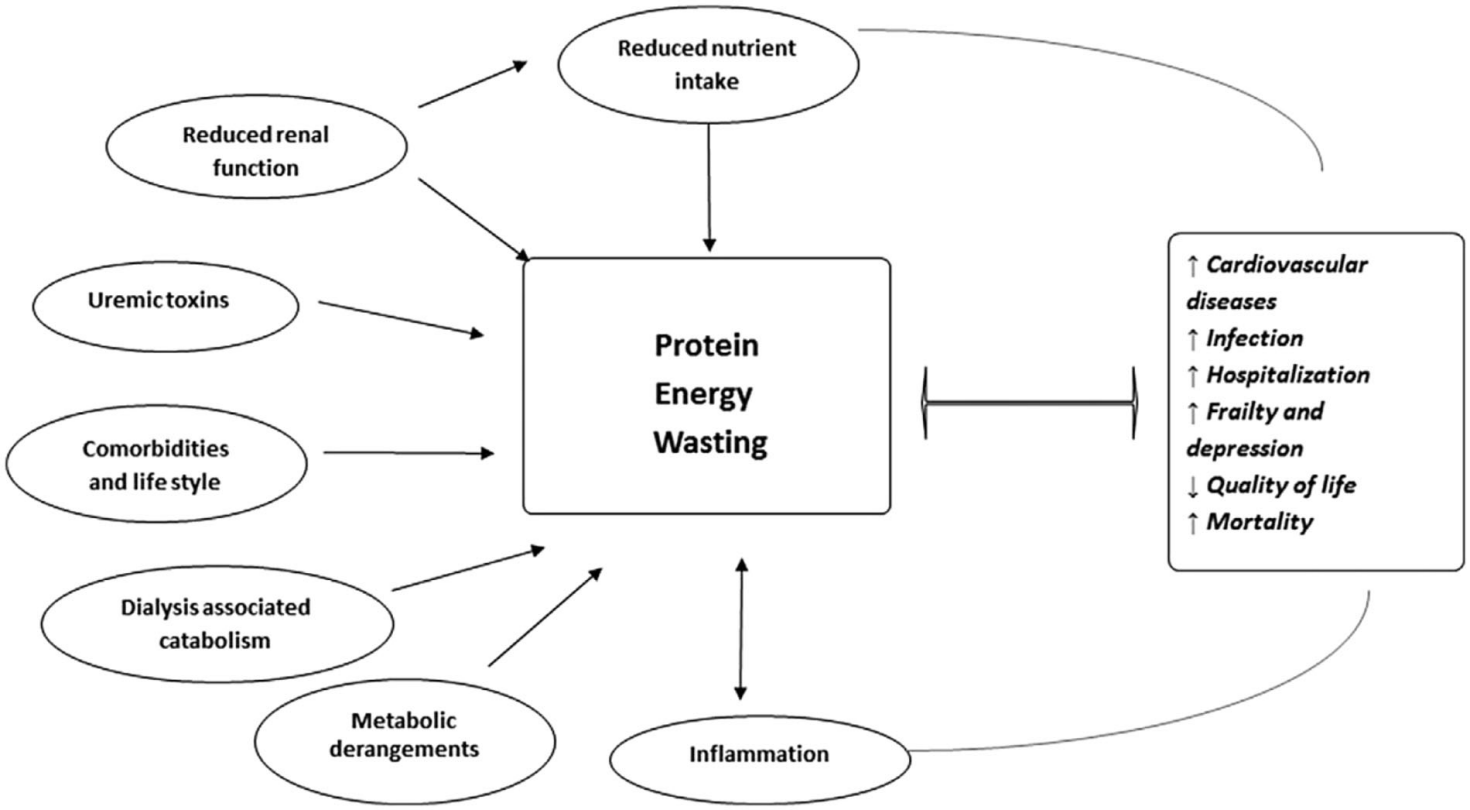
Factors that influence PEW (from Ref. [9]).

Finally, TMAO as a high burden of uremic toxin was associated with alteration of gut microbiota and disruption of the intestinal epithelial tight junction in CKD patients. The influencing factors of gut microenvironment include dietary changes, azotemia, slow colonic transit, frequent use of antibiotics, inflammation, indoxyl sulfate, and p-cresol sulfate. More than that uremia and fluid retention with bowel edema increase intestinal permeability. PEW is characterized by loss of protein and energy stores, which was influenced by those factors as shown in Figure 1 [9]. The factors are contributive to PEW development, most of which are peculiar of TMAO in CKD patients. The evidence shows that the gut microbiota-derived TMAO mainly comes from the intake of foods such as read meat, eggs, cheese, cod, algae, peanut, and soybeans, which should be asked to reduce as it is rich in amino acids that increase the production of toxin by intestinal flora. It is important to ensure that there is adequate protein intake for patients with CKD to prevent PEW, the major part of daily protein requirements should be avoided from the above foods as much as possible. Therefore, the paper by Hu et al. [1] should include the above important impact indicators to adjust for the relevance between TMAO and PEW, which can prevent the occurrence of unnecessary offset.
